# Maternal and Neonatal Behaviour in Italian Mediterranean Buffaloes

**DOI:** 10.3390/ani11061584

**Published:** 2021-05-28

**Authors:** Lydia Lanzoni, Matteo Chincarini, Melania Giammarco, Isa Fusaro, Alessia Gloria, Alberto Contri, Nicola Ferri, Giorgio Vignola

**Affiliations:** 1 Facoltà di Medicina Veterinaria, Università degli Studi di Teramo, Loc. Piano d’Accio, 64100 Teramo, Italy; llanzoni@unite.it (L.L.); mgiammarco@unite.it (M.G.); ifusaro@unite.it (I.F.); agloria@unite.it (A.G.); acontri@unite.it (A.C.); gvignola@unite.it (G.V.); 2 Istituto Zooprofilattico Sperimentale dell’Abruzzo e del Molise ‘G. Caporale’, Campo Boario, 64100 Teramo, Italy; n.ferri@izs.it

**Keywords:** maternal behaviour, neonatal behaviour, animal welfare, farm animals, buffaloes, ruminants

## Abstract

**Simple Summary:**

A clear understanding of species-specific natural behaviours is crucial for assessing animal welfare. In this regard, one of the most sensitive and critical moments for dairy farming animals is the post-partum period. Currently, very little information is available on this period in buffaloes. Therefore, the aim of the study was to investigate the maternal and neonatal behavioural patterns of Italian Mediterranean buffaloes during the early post-partum period and the relationship of both dam and calf welfare indicators (e.g., behaviours and growth rate). Behavioural patterns (postural, grooming, maternal rejection, and feeding) are described in detail for both dams and calves. Maternal rejection behaviours (pawing the calf, pushing the calf, and avoidance behaviour) are found to be negatively correlated with calf growth. In this regard, monitoring buffaloes’ maternal behaviours seems to be crucial, especially for primiparous mothers, in order to prevent welfare consequences for newborns when raised together.

**Abstract:**

The aim of this study was to describe the neonatal and maternal behaviour of Italian Mediterranean buffaloes. Thirty primiparous buffaloes were moved into individual pens 12.5 (±2.5) days before calving. Maternal and neonatal behaviours were recorded for 48 h after calving and the analysis was performed in continuous sampling with the software BORIS. Calves’ clinical evaluations (temperature, weight, and heart and respiratory rates) were performed at different time intervals and correlated with behavioural data from the dam. Data were analysed with parametric and non-parametric methods after controlling their distribution. The maternal behavioural pattern found highlighted buffaloes’ priorities during the post-partum period: firstly, they stand and start grooming to ensure proper care for the calf; it is only after this that they dedicate time to maintenance behaviours (feeding and lying). The dams mainly groomed the calf during the first six hours after calving (average time in the 1–6-h interval: 7.7 ± 2.5 min., F = (2.5, 60.2) = 75.0; *p* < 0.001) to ensure the formation of the mother–infant bond; thereafter, the behaviour decreased over time. As reported in the literature, inexperienced mothers could sometimes delay the calf’s first suckling with aggressive or rejection behaviours. In this regard, 16 buffalo dams showed at least one maternal rejection behaviour, which was found to negatively correlate with calves’ daily weight gain (DWG) at 14 (*r_s_* = −0.5, *p* = 0.02) and 21 days (*r_s_* = −0.7, *p* < 0.001). The calves took on average 212.0 ± 110.0 min to suckle, and this behaviour was mainly shown during the first six hours. Overall, suckling behaviour was correlated with standing: (*r_s_* = 0.6, *p* < 0.001) and walking (*r_s_* = 0.9, *p* < 0.001). The calves’ live weight and DWG were consistently higher than the values reported in the literature. Our results present a detailed description of maternal and neonatal behaviour in the early post-partum period in Italian Mediterranean buffaloes. We also found that maternal rejection behaviours can negatively influence the calves’ growth. Finally, we think that such results can improve the management of buffaloes during the period around parturition.

## 1. Introduction

In Italy, buffaloes are farmed mainly for the production of milk, which is generally processed into “mozzarella cheese”, a protected designation of origin product [1]. Furthermore, recently, buffalo meat has also started to pique the interest of consumers [2]. The increasing demand for buffalo-derived products has caused a recent growth of this sector. While this has surely led to economic benefits, it has also exposed animals to environmental and physiological stress [3]. 

Animal welfare has several implications for livestock. While a universal definition is still missing [4], we refer to animal welfare as a broad term that includes different measures of mental and physical state, such as physiological, health, and behavioural indicators (a detailed review of different measures can be accessed at [5]).

In buffaloes, space restriction is a relevant issue because it can affect animals’ physical and psychological state, leading to welfare concerns [3,6]. In order to ensure milk production under market requirements, in Italy, buffaloes usually undergo out-of-season breeding [7]. This, in turn, forces the buffaloes to mate during less favourable periods [8], and this could be a source of stress as well. Additionally, it causes calves to be born during winter’s cold weather [9], therefore subjecting them to stress in their early life. Furthermore, early mother–calf relationship disruption represents another cause of emotional and physiological stress, as calves in intensive dairy production systems are generally separated from their mothers immediately after birth [10,11]. Some evidence has shown that keeping the calf together with the mother in buffalo production systems could have a good outcome on calf growth [12], without compromising milk production [13]. Such data can encourage the adoption of different farming practices—for instance, the “half-day cow-calf” contact system [14,15], which is already used in some dairy cow farms. 

A clear understanding of species-specific behaviours is crucial in order to assess animal welfare. In this regard, one of the most sensitive and critical moments for farming animals is the post-partum period [16]. This is preceded by the painful process of parturition [17,18], and a long-lasting mutual dam–calf bond is established [10].The onset of this strong bond is promoted by both the maternal behavioural response and the learning ability of the calf [10], and it is also influenced by the maternal experience [16,19]. Generally, the buffalo dam stands or remains standing immediately after calving to assist the calf, while less priority is given to maintenance behaviour (e.g., lower feed intake and resting time) [17,20]. At this point, the mother learns how to identify her calf through the process of maternal grooming, a hormone-mediated behaviour [13] that stimulates the calf’s physical and biological activities [21,22]. The calf, under the cow’s stimulus, develops a behavioural pattern aimed at succeeding in suckling the udder and consuming colostrum [19].

Very little is known about the behaviour and welfare of the Italian Mediterranean buffalo dam and calf. As previously stated, the post-partum period is a sensitive time both for the mother and the calf and deserves to be given particular attention [10,23]. 

Studies on the maternal and neonatal behaviour of buffaloes are still scarce and obtaining more knowledge will positively impact farming practices. 

The present study aims to describe the behavioural pattern of the dam and calf in the short period after calving. Secondly, we also aim to define the behavioural characteristics of the dam that can positively or negatively affect calf welfare in Italian Mediterranean buffaloes. 

## 2. Materials and Methods

### 2.1. Location and Animals and Housing

The study was performed at the experimental farm of the “Istituto Zooprofilattico Sperimentale dell’Abruzzo e del Molise” (Teramo, Italy) (coordinates: 42°41′–42°.69′ N latitude and 13°44’–13°.74′ E longitude) from December 2018 to March 2019. Thirty Italian Mediterranean buffalo heifers (*Bubalus bubalis*, subspecies bubalis) and their calves were initially enrolled in the experiment. The animals were kept in group housing and moved into individual calving pens at 12.5 ± 2.5 days. The individual pens (3.5 × 3.5 m) allowed social and visual contact and were provided with straw for bedding. The animals were fed with commercial concentrate and hay, and they had ad libitum access to automatic drinkers. All the dams were artificially inseminated at the age of 20 ± 1 months. The calving day was predicted through ultrasonography, clinical evaluation of the premonitory signs, and the use of intravaginal devices, as described by Rossi et al. (2020) [24]. The latter allowed us to perform a precise assessment of the calving time, provide initial care to the newborn calves (umbilical cord disinfection), and assess clinical parameters.

### 2.2. Clinical Monitoring of Calves

Only animals born after euthocic parturition were included in this study. The birth of the calf was defined as the completion of stage 2 of the calving, which is the time between the rupture of the chorioallantois and the complete passage of the foetus.

The weight of the calves was assessed immediately after birth and at 3, 7, 14, and 21 days after parturition with a digital weighing scale.

Furthermore, immediately after, 1 h after, 12 h after, and 72 h after the birth of the calf, the following parameters were measured while gently restraining the subject: transrectal temperature (with a digital thermometer), heart rate (using a stethoscope), and respiratory rate (by visual observation). The transrectal temperature was also evaluated at day 7, 14, and 21 days (day 0 = birth). These data have already been published by Gloria et al. (2020) [23]. All the procedures were performed by a veterinarian experienced with this species.

### 2.3. Behavioural Monitoring

Since the focus was on both the dam and the calf, birth and calving terms are used to refer to the moment where the calf was completely expelled (when not explicitly mentioned otherwise).

Animal behaviour was continuously video monitored using 4 infrared cameras (CCTV colour camera; AHD-40A130 model; Sony). Each camera framed two pens at the same time and was connected to a computer that stored the video. The videos obtained were downloaded from the computer, selecting the interval between the birth of each calf until 48 h after birth and then imported into the software Behavioural Observation Research Interactive Software (BORIS) [25]. The ethograms used for the study are reported in Table 1. The behaviours were divided into different categories: postural, grooming, self-grooming, feeding, social, and maternal rejection behaviours (MRBs). The behaviours were recorded as either events (behaviour without duration but only frequency) or states (behaviour with both duration and frequency). “Interference” time (when at least one operator was inside the pen) and the time where the animals were not visible were summed and labelled as “not visible”. Finally, the behavioural analyses were carried out using continuous sampling by the same operator. 

### 2.4. Statistical Analyses

The behavioural data were exported in time and percentage formats from BORIS for the following time periods after calving: hourly for the first 6 h, 6-h interval for the first 24 h, and daily total amount of time for the first and second day. To assess the percentage of time displaying a specific behaviour, the “not visible” time was subtracted from the total time using BORIS. All the statistical analyses were performed using the software Jamovi [30]. All the parameters were reported in minutes or frequencies as means ± standard deviation (SD) and, for some values, the interval (minimum–maximum) was also reported. Data were tested for normality with the Shapiro–Wilk test and checked visually with a Q-Q plot. The following data were transformed to meet normality assumptions (type of transformation (data-time interval)): natural logarithm (Dam-Walking-daily variables), squared roots (Dam-Grooming-daily, six-hour and hourly variables, Calf-Sniffing-daily variables), and box cox (Calf-Lying-daily variables). The behavioural variables for both dams and calves were evaluated considering several time intervals: total time (48 h after birth), the first day (0–24 h), and the second day (25–48 h) after calving. Furthermore, the 6-h time intervals (0–6 h, 7–12 h, 13–18 h, 19–24 h) and hourly intervals during the first 6 h were analysed separately. Behavioural differences between days were assessed with a *t*-test for repeated measures (Student T for data with a normal distribution and Wilcoxon for data with a non-normal distribution). According to the literature, most of the behavioural changes in both the dam and calf occur within the first six hours after calving [19,20]. Due to this, and in order to make a more reliable comparison with previous results, a repeated-measures ANOVA or Friedman test were performed for the six-hour and hourly intervals. Tukey’s test was used as a post hoc test for the ANOVA, and the Durbin–Conover test was performed for Friedman’s test. In order to see if calving time influenced the behavioural outcomes, the animals were grouped according to the calving time: day-time calving (from 6:00 a.m. to 6:00 p.m.) and night-time calving (from 6:00 p.m. to 6:00 a.m.). To assess the difference in the total time (48 h) between the calves’ sexes and calving time, an independent sample *t*-test (Student T or Mann–Whitney, according to the data distribution) was carried out. The relationship between behavioural and clinical variables (weight and daily weight gained) was assessed using Pearson’s (r_p_) or Spearman’s (*r_s_*) correlation according to the data distribution, and results with *p* < 0.05 were considered statistically significant. A flow diagram of calf behaviours was created with Graphviz (Graph Visualization Software; [31]) to verify the dynamic of the calf behavioural pattern after birth from the frequencies of the transition matrix exported from BORIS.

## 3. Results

Five primiparous dams and four calves were later excluded from the study for the following reasons: one heifer had a spontaneous miscarriage, while technical problems occurred in the video recording for the others. Therefore, the final number was 25 heifers and 25 calves (11 females and 14 males). Even though the dam drinking behaviour was initially included in the ethogram, the results were not reliable, since in most of the cases animals were only partially visible and removed from further analysis. When more than 80% of the behaviour was not visible because of the blinded corner of the camera, subjects were excluded from the analysis (as reported in Table 2).

### 3.1. Maternal Behaviours

#### 3.1.1. Latency Time between Calving and the First Behavioural Activities

The latency time from calving to the first behavioural activities is reported in Table 2. Fourteen dams gave birth standing and so were excluded from the latency to stand evaluation. A positive correlation between the latency from calving to grooming and from calving to standing was identified (*r_s_* = 0.7, *p* = 0.001).

#### 3.1.2. Postural Behaviours (Standing and Walking)

No significant differences were found between the time spent lying and walking during the first two days after calving (Table 3). Considering the 6-h intervals, the average lying time during the 0–6-h interval was significantly lower than that of all the other intervals, while no significant difference was found between the intervals for walking time (Table 4). Considering the hourly variables, the dams tended to spend significantly less time lying during the first two hours after calving. The dams tended to walk for less time during the first hour compared with the second to the fifth hour after calving; in contrast, no difference was found between the first and sixth hour (Table 5).

#### 3.1.3. Feeding Behaviour

A paired sampled *t*-test showed that the buffaloes tended to spend less time feeding during the first day than during the second day after calving (Table 3). No significant difference was identified between the six-hour intervals and the hourly variables. 

### 3.2. Calves’ Clinical Parameters

The mean values for the weight and daily weight gain (DWG) are reported in Table 6. No significant differences were identified in either the weight or DWG between sexes. 

### 3.3. Calves’ Behaviours

#### 3.3.1. Latency between Birth and the First Behavioural Activities

**Figure 1:**
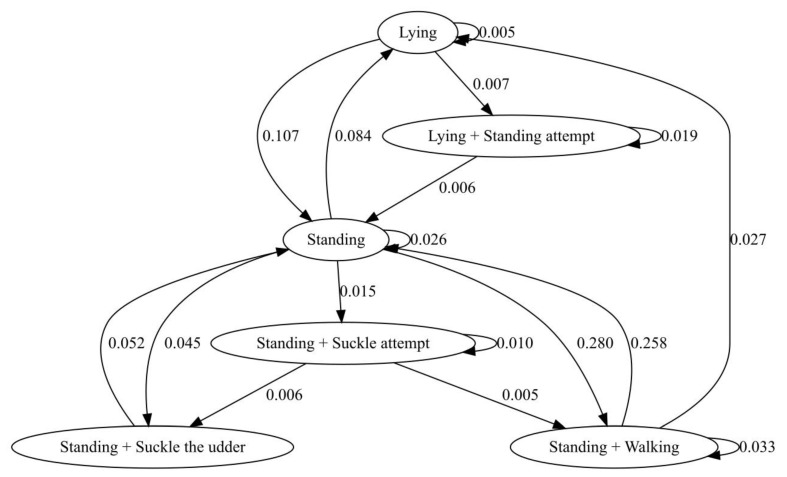
Transition flow diagram of the calves’ behaviours in the first 48 h after birth. The values represent the frequency of the transitions between the different behaviours. The diagram was obtained from the transition matrix exported from BORIS selecting the following behaviours: lying, standing, standing attempt, suckling attempt, walking, and suckling the udder. The final diagram was obtained from Graphviz, selecting the behaviours with a frequency of transition higher than 0.005.

**Figure 2:**
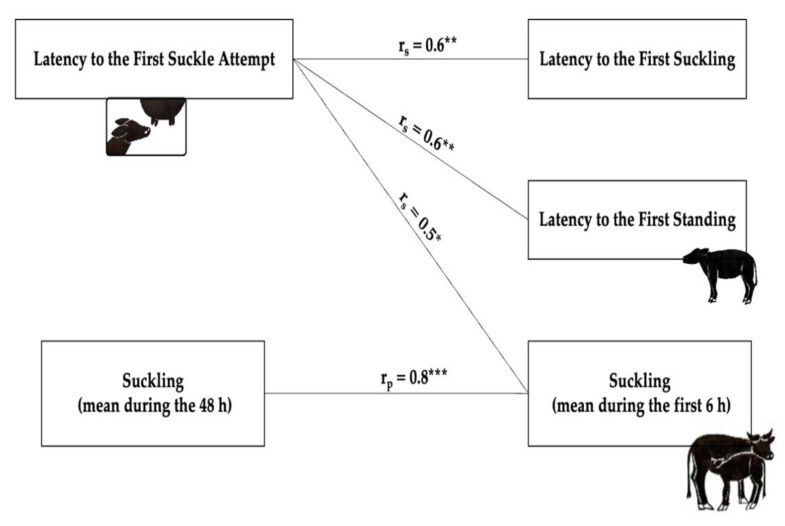
Correlations between different calves’ behaviours. *r_s_* = Spearman’s correlation; *r_p_* = Pearson’s correlation. * *p* < 0.05; ** *p* < 0.01; *** *p* < 0.001.

Table 2 displays the mean latency from birth to the first behavioural activities in calves. The calves born during the night tended to take significantly more time (160.0 ± 74.9 min) to suckle for the first time compared with the calves born during the day (132.0 ± 74.9 min) (U = (N*_night_* = 14, N*_day_* = 10) = 36.0, *p* = 0.048, and rank biserial correlation = 0.5). Despite the differences, all the calves suckled for the first time within eight hours after birth; thus, no assistance was needed. The behavioural pattern of the calves during the first 48 h of life is reported in the transition flow diagram (Figure 1). Immediately after birth, the calves were lying, then they started making their first attempts to stand up until they managed to stand up successfully. At this point, they started making attempts to suckle until they finally succeeded. As reported in Figure 2, positive correlations were identified between the latency to the first suckling attempt and the latency to the first successful suckling (*r_s_ =* 0.6, *p* = 0.003) and the first successful standing (*r_s_* = 0.6, *p* = 0.005), while a negative relationship was found with the average suckling time during the 6 h after birth (*r_s_* = 0.5, *p* = 0.03).

#### 3.3.2. Postural Behaviours

The calves spent more time lying during the second day after birth than during the first day (Table 3). Considering the six-hour intervals, the calves tended to spend less time lying during the first six hours after birth, with a slow increase in the behaviour in the following intervals (Table 4). A comparison between the hourly intervals showed that the calves tended to spend significantly less time lying during the first five hours after birth when compared with the sixth hour (Table 5). A positive correlation was found between the average walking time during the 48 h (1.1 ± 0.6 min/h) and the average standing time (8.5 ± 2.7 min/h; *r_s_* = 0.5, *p* = 0.007).

The calves walked for 36.7 ± 22.1 min during the first day after birth and for a significantly shorter amount of time during the second day (Table 3). Considering the six-hour intervals, the calves walked significantly more during the first six hours after birth when compared with the other intervals. A general slowly decreasing tendency was identified between the six-hour time intervals (Table 4). Looking more specifically into the first six hours, a significantly shorter amount of time was spent walking during the first hour after calving compared with the following hours (Table 5). 

#### 3.3.3. Suckling

The buffalo calves were observed suckling for a significantly longer amount of time during the first day than during the second day after calving (Table 3). The calves tended to suckle the udder for a significantly longer amount of time during the first six-hour interval (and particularly in the first five hours), with a tendency towards reducing the time dedicated to this behaviour over time (Table 4 and Table 5). The mean suckling bout duration was 3.1 ± 1.0 min, and the suction frequency during the 48 h after birth was 24.0 ± 13.6 events per calf. A positive correlation was identified between the mean suckling time during the 48 h (1.2 ± 0.7 min/h), the mean standing time (8.5 ± 2.7 min/h; *r_s_* = 0.6, *p* < 0.001), and the average walking time (1.1 ± 0.6 min/h; *r_s_* = 0.9, *p* < 0.001).

### 3.4. Dam–Calf Interactions

#### 3.4.1. Buffaloes’ Grooming Behaviour 

From the videos, it was difficult to distinguish between the sniffing and licking time displayed by the mother, so these two behaviours were reported combined as grooming behaviour. The buffalo dams tended to groom their calves more during the first day than during the second one after calving (Table 3). The mean duration of grooming during the first six hours was significantly higher than in the other intervals, where a decreasing tendency was observed (Table 4). More specifically, the dams tended to manifest the grooming behaviour more during the first hour after calving, with a highly significant decrease from the first to the second hour. The behaviour started to decrease gradually from the first to the sixth hour, with a slight increase during the fourth hour after calving (Table 5). 

#### 3.4.2. Calves’ Sniffing Behaviour

The calves tended to sniff the dams for a significantly higher amount of time during the first day as compared with the second day after birth (Table 3). The sniffing behaviour was observed for a significantly lower amount of time during the first six hours after birth than during the other six-hour intervals (Table 4). However, after the first hour, the calf increased the sniffing behaviour (Table 5).

#### 3.4.3. Maternal Rejection Behaviour

An average frequency of 4.7 ± 9.1 MRBs was manifested by the buffalo dams during the first day after parturition, while a significantly lower number was displayed during the second day. The most frequently expressed behaviour among the MRBs was the avoidance one (Table 3). Considering the 6-h intervals, a significantly higher number of MRBs was manifested during the 1–6-h intervals. When considering the hourly intervals, a significantly higher number was manifested during the second hour after parturition (Table 5). Among the buffalo dams, 16 expressed at least one MRB, 12 of them showed between 0 and 10 MRBs, one buffalo dam showed between 10 and 20, one showed between 20 and 30, and two showed between 20 and 40. A positive correlation between the number of MRBs manifested and the mean dam lying behaviour during the 48 h (30.4 ± 3.6 min) was also found (*r_s_* = 0.5, *p* = 0.012).

#### 3.4.4. Further Considerations on Maternal Behaviours and Calves’ Growth

A negative correlation was found between the number of MRBs expressed during the 48 h (5.3 ± 9.8) and the daily weight gain at 14 (*r_s_* = −0.5, *p* = 0.02) and 21 days after calving (*r_s_* = −0.7, *p* < 0.001). Moreover, a negative correlation was found between the average lying time of the dam during the 48 h and the daily weight gain at 21 days (*r_p_* = −0.5, *p* = 0.02). The dams’ average grooming time (2.6 ± 0.8 min) was positively correlated with the calves’ average standing time (8 ± 2.7 min; *r_p_* = 0.5, *p* = 0.01) and the average sniffing time during the 48 h (1.8 ± 1.1 min; *r_s_* = −0.6, *p* = 0.001).

## 4. Discussion

The present study aimed to define the behavioural pattern of the dam and calf after calving (I) and to define the dam’s behavioural characteristics that could positively or negatively affect calf welfare in Italian Mediterranean buffaloes (II). Due to the scarcity of research on buffalo behaviour, most of the references used for discussing the results refer to dairy cows, since these were considered the most proximate species useful for comparisons.

The first hours of the immediate post-calving period represent a critical period for both the dam and the calves: the mother tends to stand up immediately to dedicate proper care and allow colostrum suckling to the calf [29]. We found that buffaloes took longer to stand up than was reported by Jensen (2012) [20] for cows but shorter than that reported for heifers [32,33]. Almost half of the dams involved in our study gave birth standing, different to what was reported by other authors in cows [19], sheep [34], and wild ungulates [35]. Selman et al. (1970) [29] observed that two thirds of the cows completed calving standing; in our case, half of the dams stood while completing the expulsion of the calf.

Dams were observed lying less during the first hour and this behaviour increased with time, as reported previously in both buffaloes and cows [17,19,20]. In contrast with Jensen (2011) and Huzzey et al. (2005) [18], no difference was found in the lying time of the mother from the first to the second day after calving. This could mean that buffalo dams compensated rapidly for the high activity of the first hours after birth. In general, we observed dams lying for a shorter amount of time compared with dairy cows and Surti buffaloes [17,19,20], suggesting that there was a species-specific difference in the Italian Mediterranean buffaloes. 

After standing up, the dams started licking or sniffing the calf with a latency time comparable with the results of Vandenheede et al. (2001) [36] but higher than that reported by other authors [20,33]. No failure in calf grooming was observed, different from the previous results [19,36,37]. The proximity in latency to standing and grooming has been previously described in bovines by Selman et al. (1970) [29], and the positive correlation identified between these two variables suggests that dams that stand first also start to groom first [17]. Grooming behaviour is mediated by hormonal changes [13]; it stimulates the calf’s general activity [21,22] and contributes to the creation of a strong cow–calf bond [38]. This behaviour was mainly displayed during the first six-hour interval and then reduced gradually, in agreement with previous studies [17,19,28,33,39]. During the first six hours, the buffaloes groomed for an amount of time comparable with the results from Edwards and Broom (1982) in dairy cows [19]. However, these were shorter than those reported by Jensen (2012) in dairy cows [20] and Dubey et al. (2018) in buffaloes [17]. A possible explanation for these differences could be linked with the maternal experience of the dams, since less-experienced mothers could tend to express this behaviour less [16,19] and our sample comprised only primiparous buffaloes. A significant reduction in this behaviour from the first to the second hour was observed, followed by a small but still significant decrease in the following hours, as seen in dairy cows [19]. Usually, animals tend to groom mainly throughout the time of the formation of the mother–infant bond, when the stimuli of birth fluids are more intense. The decrease in the manifestation of grooming could be due to the desaturation of the neonatal coat of amniotic fluids and to changes in the characteristic of the stimulus from the calf [32]. The correlation found between maternal grooming and calf standing confirms the role of maternal licking in the stimulation of calves’ nervous systems [32]. However, it is difficult to differentiate between the cause and the effect, since the calf’s activity could also stimulate the dam’s licking behaviour [32]. In any case, this correlation strengthens the hypothesis of a reciprocal mother–calf influence on the manifestation of the behaviours.

The dam’s behavioural pattern indicates buffaloes’ priorities during the post-partum period: firstly, they stand and start grooming to ensure proper care to the calf; it is only after this that they dedicate time to maintenance behaviours, such as feeding or lying. Compared to dairy cows, the latency to feeding was longer [19] and the feeding trend (the amount of time spent in feeding behaviour during the six-hour interval) was higher than what was reported by Jensen (2012) [20]. On the other hand, the feeding behaviour trend was consistent with the results of Dubey et al. (2018) [17], considering the differences in the sampling time in buffaloes. Moreover, no significant variation was noted during the first day, differently from Jensen (2012), but a slight increase from the first to the second day was observed. This could be explained by the fact that even if buffaloes prioritised the calves’ care during the first day, they also tried to keep a balance between maternal and maintenance behaviour.

In general, the mother should facilitate the calf’s teat-seeking behaviour, but inexperienced mothers could tend to show a delay in the expression of maternal care [10,16,20,27] or could delay the access of the offspring to the udder [19,29,40,41] with aggressive or rejection behaviours. MRBs were occasionally registered, as previously described by Edwards and Broom (1982) [19] and Selman et al. (1970) [29], but did not completely inhibit the calves from suckling. The number of avoidance behaviours manifested during the first six hours was comparable to what was reported by Edwards and Broom (1982) [19] in heifer cows. The manifestation of MRBs was synchronised with the time the calves started to interact with their mothers. MRBs and dam lying time were negatively correlated with DWG at the 14th and 21st day. The latter suggest that mothers avoiding active or passive interaction with their calves negatively influence their normal behaviour and postnatal growth. This underlines how the behaviours and the maternal attitude should be monitored by farmers, especially in primiparous buffaloes in the first days post-partum.

The calf behavioural pattern observed is consistent with the one shown in the literature: the calf raises its head, places itself in a ventral-sternal position, and starts attempting to stand until it manages to stand successfully. Then, it approaches the udder to begin suckling [10,17]. The latency from birth to the first standing attempt was longer compared with the value from the literature on buffaloes [42] and cows [20,39]. The latency from birth to the first successful standing agreed with the observations of Houwing et al. (1990) [33] but was longer than that seen in other studies in cows [19,20,36,39,43]. The number of standing attempts made by the calves was similar to that reported by Houwing et al. (1990) [33] and Ventorp and Michanek (1991) [43] in cows but smaller than that reported by Dubey et al. (2018) [17] in buffaloes.

In accordance with the results of Jensen (2012) [20] in dairy cows, the calf lay for less time during the first day, especially during the first six hours. In fact, during the first hours there was an onset of the calf’s exploratory activities and all the behaviours were teat-seeking oriented [19]. This is also highlighted by the peak of the number of standing and suckling attempts reached during the first and the second hour, respectively.

Compared to other studies on buffaloes, the number of suckling attempts was similar while the latency to suckle observed was longer [17] than that in dairy cows [33,36]. We found that calves that were born during the night-time took longer to suckle for the first time. However, this did not represent a risk for the colostrum intake, as the calves were suckling within three hours. In contrast to what has been observed in bovines, a lower percentage of calves (8%) did not manage to suckle within six hours [19,37]. Differently from Vandenheede et al. (2001) [36] and consistent with Lidfors (1996) [39], a difference in the suckling behaviour during the first two days was found, with calves suckling more during the first day. The suckling duration was in agreement with Jensen (2012) but lower than that reported by Edwards and Broom (1982) [19] during the first six hours. The calves suckled more during the first six hours, hitting a peak between the third and the fifth hours.

The correlations found between the latencies suggested that calves that took more time to stand also took more time to make the first suckling attempt, as previously reported in bovines by Campler et al. (2015) [27]. Similarly to Dubey et al. (2018) [17], calves that took more time to make the first suckling attempt took more time to suckle for the first time, and in our case, they also suckled less during the first six hours after calving. Moreover, the correlation found between the suckling and the postural behaviours (standing and walking) could be related to the calf’s vitality. 

The live weight and daily weight gain of the calves involved in our study were consistently higher than those reported in the literature for Italian Mediterranean buffalo calves by various authors [6,9,44,45]. This could suggest that allowing the calf to stay with the dam could be beneficial for calf weight gain, as reported by several authors for cows [46,47,48,49,50] and buffaloes [12]. In addition to this, Singh et al. (2017) [13] found that keeping the calf and dam together could also have a beneficial effect on the milking procedures, reducing the milking let-down time and improving the flow rate and quantity of milk. Finally, we want to highlight that the behavioural differences could also partially be explained by the diversity in the experimental designs among the studies mentioned. 

## 5. Conclusions

Italian Mediterranean buffalo farming is undergoing a process of intensification, and more information regarding the behaviour and welfare of this species is required. One of the most critical phases in dairy production is represented by the disruption of the maternal–filial relationship. We described in detail the maternal behaviour after calving and how some aspects can influence calf welfare. Such results can help to improve the management of buffaloes during calving time. In particular, we want to underline the importance of preventing welfare consequences on newborns by monitoring maternal behaviours, since signs related to maternal rejection behaviour could also affect weight gain in healthy calves born after parturition.

Even if this was out of the scope of the study, we want to stress the need for further research considering production systems where the calf can spend the first days after birth with the mother. An open question remains of whether these systems could indeed have positive consequences for both the calf’s health and welfare.

## Figures and Tables

**Table 1:** Ethogram of the behaviour observed for the dam and the calf.

	Category	Description	Recording Event (e) or State (s)
**Dam**			
Lying	Postural	Lying on the sternum or on the side. Head may be rested or raised.	S
Standing	Postural	Body supported by four legs, standing still, or standing and walking.	S
Walking	Postural	Body supported by four legs, making at least two steps forward or backwards.	S
Licking the calf	Grooming	Tongue in contact with the calf’s head or body.	S
Sniffing the calf	Grooming	Muzzle in contact with, or in the proximity with, the calf’s head or body.	S
Pawing the calf	Maternal Rejection Behaviours	The dam raises and touches the calf with the foreleg.	E
Pushing the calf	Maternal Rejection Behaviours	The dam presses the calf down or away with the head.	E
Avoidance behaviour	Maternal Rejection Behaviours	Any movement made by the dam to move away when the calf tries to approach the udder.	E
Feeding	Feeding	Head in the feeding trough, or over the feeding trough while chewing.	S
Drinking	Feeding	Muzzle in drinking bow.	S
**Calf**			
Standing attempt	Postural	The calf is partially standing upright with both hind legs extended and with front legs still underbody but without reaching a fully upright position.	E
Lying	Postural	Lying on the sternum or on the side. Head may be rested or raised.	S
Standing	Postural	Body supported by four legs, standing still, or standing and walking.	S
Walking	Postural	Body supported by four legs, making at least two steps forward or backwards.	S
First successful standing	Postural	The calf is standing upright with all 4 legs fully extended for longer than 5 s.	E
Sniffing the dam	Grooming	Muzzle in contact with, or in close proximity with, the dam’s muzzle, or head, or body.	S
Suckle attempt	Feeding	While standing, the calf is positioned below the standing dam with the head located under the front of the dam’s body.	E
Suckle udder (successful)	Feeding	While standing, the calf is positioned below the standing dam with head located at the udder.	S

Adapted from Barrier et al. (2012) [26], Campler et al. (2015) [27], Jensen (2011) [28], and Selman et al. (1970) [29].

**Table 2:** Latency between birth and the first behavioural activities for the calf and the dam.

**Calf**	**N°**	**Mean ± SD**	**Range**
Birth-Standing (min.)	24 *	77.0 ± 47.5	27.9–214.0
Birth-Suckling (min.)	24 *	212.0 ± 110.0	64.0–439.0
Birth-Standing attempt (min.)	22 *	45.2 ± 46.3	5.5–197.9
Birth-Suckling attempt (min.)	24 *	152.0 ± 91.1	48.8–364.0
Standing attempts (n°)	25	9.2 ± 16.4	0.0–81.0
Suckling attempts (n°)	25	11.8 ± 9.5	1.0–41.0
**Dam**			
Calving-Standing (min.)	11 *	25.2 ± 17.1	0.3–55.9
Calving-Grooming (min.)	25	11.8 ± 15.0	0.0–45.0
Calving-Feeding (min.)	25	41.9 ± 23.3	3.9–107.0

* Missing subjects with more than 80% of their behaviour not visible.

**Table 3:** Behaviours during the first two days after calving.

	Days after Calving						
	1		2				
	Mean	SD	Mean	SD	*t*-Test/Wilcoxon	*p*-Value	Effect Size
**Dam**							
Visible time (min)	1342.4	±84.0	1361.3	±51.4			
Lying (min)	716.4	±121.9	737.6	±90.6	*t* (24.0) = −0.9	0.4	
Walking (min) a	33.6	±16.7	37.9	±16.8	*t* (24.0) = −1.9	0.1	
Grooming: licking + sniffing (min) b	88.0	±25.0	36.1	±19.0	*t* (24.0) = 12.1	<0.001	2.4 *
Feeding (min)	280.0	±45.4	306.0	±55.7	*t* (24.0) = −2.5	0.02	−0.5 *
Maternal Rejection behaviours (n°)	4.7	±9.1	0.6	±1.6	W = 127.0	0.003	0.9 ¥
Pawing the calf (n°)	0.0	±0.0	0.0	±0.2			
Pushing the calf (n°)	0.1	±0.4	0.0	±0.0			
Avoidance behaviour (n°)	4.6	±9.1	0.6	±1.6			
**Calf**							
Visible time (min)	1332.0	±135.4	1339.4	±260.0			
Lying (min) c	1089.5	±132.4	1174.6	±235.8	*t* (24.0) = 12.4	<0.001	2.5 *
Standing attempts (n°)	11.9	±16.6	0.1	±0.3	W = 325.0	<0.001	1.0 ¥
Walking (min)	36.7	±22.1	18.0	±8.9	*t* (24.0) = 4.9	<0.001	1.0 *
Sniffing (min) b	55.8	±41.5	28.0	±20.1	*t* (24.0) = 5.0	<0.001	1.0 *
Suckling attempts (n°)	10.6	±8.8	1.2	±2.4	W = 300.0	<0.001	1.0 ¥
Suckle the udder (min)	38.0	±22.6	21.9	±13.5	*t* (24.0) = 4.1	<0.001	0.8 *

Number of subjects: 25; effect size: * = Cohen’s d; ¥ = Rank biserial correlation. Data transformations: a = natural logarithm, b = squared roots, c = box cox.

**Table 4:** Behaviours during the six-hour time intervals.

	1–6 h	7–12 h	13–18 h	19–24 h	Friedman/ANOVA Results	*p*-Value
**Dam**						
Visible (min/h)						
Lying (min/h)	21.9 ± 6.8-A	34.4 ± 7.4-B	31.2 ± 10.3-B	31.9 ± 9.2-B	F (3.0, 7.0) = 11.8	<0.001
Grooming (min) a	7.7 ± 2.5-A	2.6 ± 1.1-B	2.5 ± 1.4-BC	1.9 ± 1.4-C	F (2.5, 60.2) = 75.0 **	<0.001
Feeding (min)	11.2 ± 4.3	11.3 ± 4.6	11.4 ± 2.7	12.8 ± 4.9	χ^2^ (3.0) = 3.5	0.3
Walking	1.4 ± 1.0	1.2 ± 1.0	1.5 ± 1.4	1.6 ± 1.0	χ^2^ (3.0) = 3.0	0.4
Maternal Rejection Behaviours (n°)	0.7 ± 1.5-A	0.0 ± 0.14-B	0.0 ± 0.1-B	0.0 ± 0.1-B	χ^2^ (3.0) = 33.1	<0.001
**Calf**						
Visible (min/h)	50.6 ± 7.9	58.0 ± 2.4	56.9 ± 5.3	56.4 ± 10.6		
Lying (min/h)	33.9 ± 8.5-A	48.2 ± 6.5-B	49.8 ± 6.3-B	49.7 ± 10.7-B	χ^2^ (3.0) = 38.7	<0.001
Standing attempts (n°/h)	1.7 ± 1.8-A	0.2 ± 1.0-B	0.1 ± 0.2-C	0.0 ± 0.1-C	χ^2^ (3.0) = 57.7	<0.001
Suckling the udder	2.8 ± 2.2-A	1.7 ± 1.7-B	1.1 ± 0.8-B	0.7 ± 0.7-C	χ^2^ (3.0) = 25.9	<0.001
Suckling attempts (n°/h)	1.4 ± 1.4-A	0.2 ± 0.2-B	0.2 ± 0.4-B	0.1 ± 0.1-B	χ^2^ (3.0) = 41.6	<0.001
Sniffing the dam (min/h)	4.3 ± 3.2-A	1.9 ± 1.8-B	1.2 ± 0.9-B	1.9 ± 4.6-B	χ^2^ (3.0) = 22.0	<0.001
Walking (min/h)	2.8 ± 2.2-A	1.7 ± 1.6-B	1.1 ± 0.8-B	0.7 ± 0.7-C	χ^2^ (3.0) = 26.7	<0.001

Number of subjects: 25; mean times of the behaviours were compared with an ANOVA or Friedman test (the corresponding *p* value is reported in the table). Data transformations: a = squared roots. Within rows, values with different letters (A, B, C, D) were significantly different (*p* < 0.05) at the post hoc comparison (Tukey for ANOVA and Durbin–Conover for Friedman). ** (Huynh–Feldt correction).

**Table 5:** Behaviours during the hourly intervals in the first six hours after calving.

	1 h	2 h	3 h	4 h	5 h	6 h	Friedman/ANOVA Results	*p*-Value
**Dam**								
Visible (min/h)	38.1 ± 12.6	50.2 ± 12.6	50.7 ± 12.3	56.5 ± 7.9	55.6 ± 8.6	56.3 ± 8.2		
Lying (min/h)	6.2 ± 10.8-A	13.4 ± 12.1-AC	20.4 ± 13.8-C	31.6 ± 16.6-B	27.7 ± 10.5-BC	32.1 ± 17.0-B	χ ^2^ (5.0) = 48.9	<0.001
Grooming a (min)	17.4 ± 9.5-A	9.4 ± 5.8 -B	6.5 ± 3.4-BC	4.2 ± 4.2-C	5.5 ± 4.6 -BC	3.2 ± 2.5-DC	F (5.0, 120.0) = 19.8	<0.001
Feeding (min)	8.0 ± 6.7	14.6 ± 7.9	10.7 ± 7.1	10.6 ± 6.6	12.6 ± 11.6	10.9 ± 9.00	χ^2^ (5) = 5.8	0.3
Walking (min)	0.4 ± 0.8-A	2.1 ± 1.7 -B	2.0 ± 2.3-B	1.5 ± 1.6 -B	1.3 ± 1.3-EB	1.1 ± 1.4 -ABE	χ^2^ (5.0) = 22.9	<0.001
Maternal Rejection Behaviours (n°)	0.1 ± 0.4-A	0.9 ± 1.69 B	1.2 ± 5.2-A	0.6 ± 1.7-A	0.6 ± 1.9-AB	0.7 ± 3.2-A	χ^2^ (5.0) = 13.4	0.020
**Calf**								
Visible (min/h)	37.1 ± 13.0	48.8 ± 17.2	51.7 ± 11.3	55.2 ± 13.4	53.5 ± 14.4	57.4 ± 6.4		
Lying (min/h)	32.9 ± 14.4-A	26.0 ± 20.1-A	28.9 ± 15.2-A	35.4 ± 20.0 A	35.9 ± 17.4-A	44.3 ± 14.6-B	χ^2^ (5.0) = 15.3	0.009
Standing attempts (n°/h)	5.3 ± 4.8-A	2.3 ± 3.2-B	1.1 ± 2.6-C	0.6 ± 1.3-C	0.4 ± 1.6-C	0.3 ± 1.1-C	χ^2^ (5.0) = 56.4	<0.001
Suckling the udder (min/h)	0.5 ± 1.6 -A	1.9 ± 2.7-BD	2.9 ± 3.8-BCD	5.0 ± 5.8-C	4.1 ± 5.0-C	2.6 ± 3.7-D	χ^2^ (5.0) = 30.3	<0.001
Suckling attempts (n°/h)	0.2 ± 1.0-A	2.3 ± 3.1-B	1.7 ± 3.8-B	1.2 ± 1.8-B	1.3 ± 3.1-B	1.6 ± 3.5-B	χ^2^ (5.0) = 14.7	0.012
Sniffing (min/h)	0.8 ± 2.3-A	4.9 ± 5.9-B	5.8 ± 7.7-B	4.4 ± 5.2-B	6.6 ± 9.7-B	3.1 ± 5.0-B	χ^2^ (5.0) = 19.8	<0.001
Walking (min/h)	0.6 ± 1.7-A	2.1 ± 2.8-B	3.4 ± 3.7-BF	4.0 ± 4.3-CF	4.0 ± 5.0-DF	2.5 ± 3.3-BF	χ^2^ (5.0) = 29.4	<0.001

Number of subjects: 25; Mean time of the behaviours were compared with an ANOVA or Friedman test (the corresponding *p* value is reported in the table). Data transformations: a = squared roots. Within rows, values with different letters (A, B, C, D, E, F) were significantly different *(p* < 0.05) at the post hoc comparison (Tukey for ANOVA and Durbin–Conover for Friedman).

**Table 6:** Weight and DWG monitored at birth and at 3, 7, 14, and 21 days after birth.

		Birth (d1)	d3	d7	d14	d21
**Weight (kg)**	**Total**	40.9 ± 3.2	44.8 ± 3.2	51.4 ± 3.6	61.6 ± 4.5	72.5 ± 4.3
	**n** **°**	25	22	24	24	22
	**Female**	39.1 ± 3.3	42.9 ± 3.3	49.5 ± 3.7	60.0 ± 5.3	71.6 ± 4.4
	**n** **°**	11	9	11	11	11
	**Male**	42.3 ± 2.3	46.0 ± 2.5	52.9 ± 2.8	62.9 ± 3.4	73.4 ± 4.1
	**n** **°**	14	13	13	13	11
**Daily weight gain (kg/day)**			1.3 ± 0.7	1.5 ± 0.3	1.5 ± 0.2	1.5 ± 0.16
	**n°**		22	24	24	22

## Data Availability

The data presented in this study are available in the Supplementary Material Data Table S1.

## Supplementary Materials

The following are available online at https://www.mdpi.com/article/10.3390/ani11061584/s1, Data Table S1.
